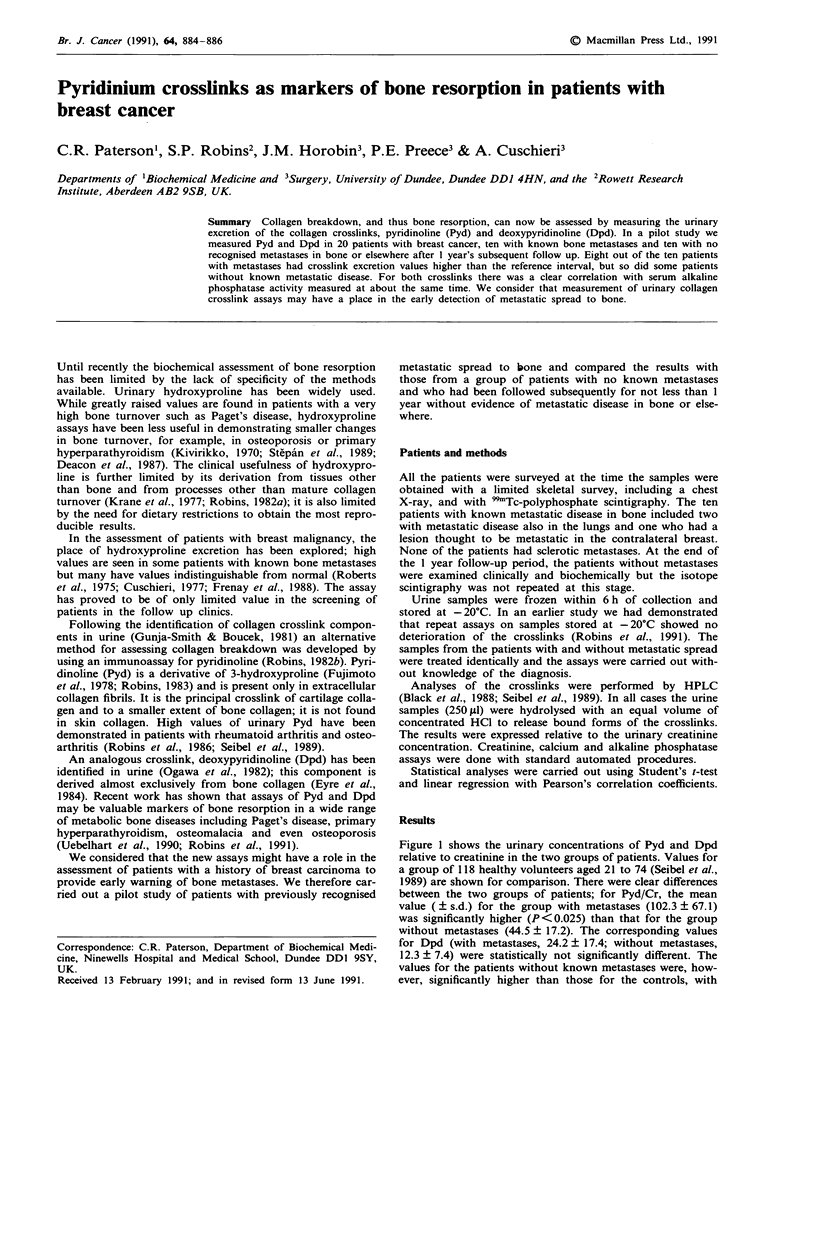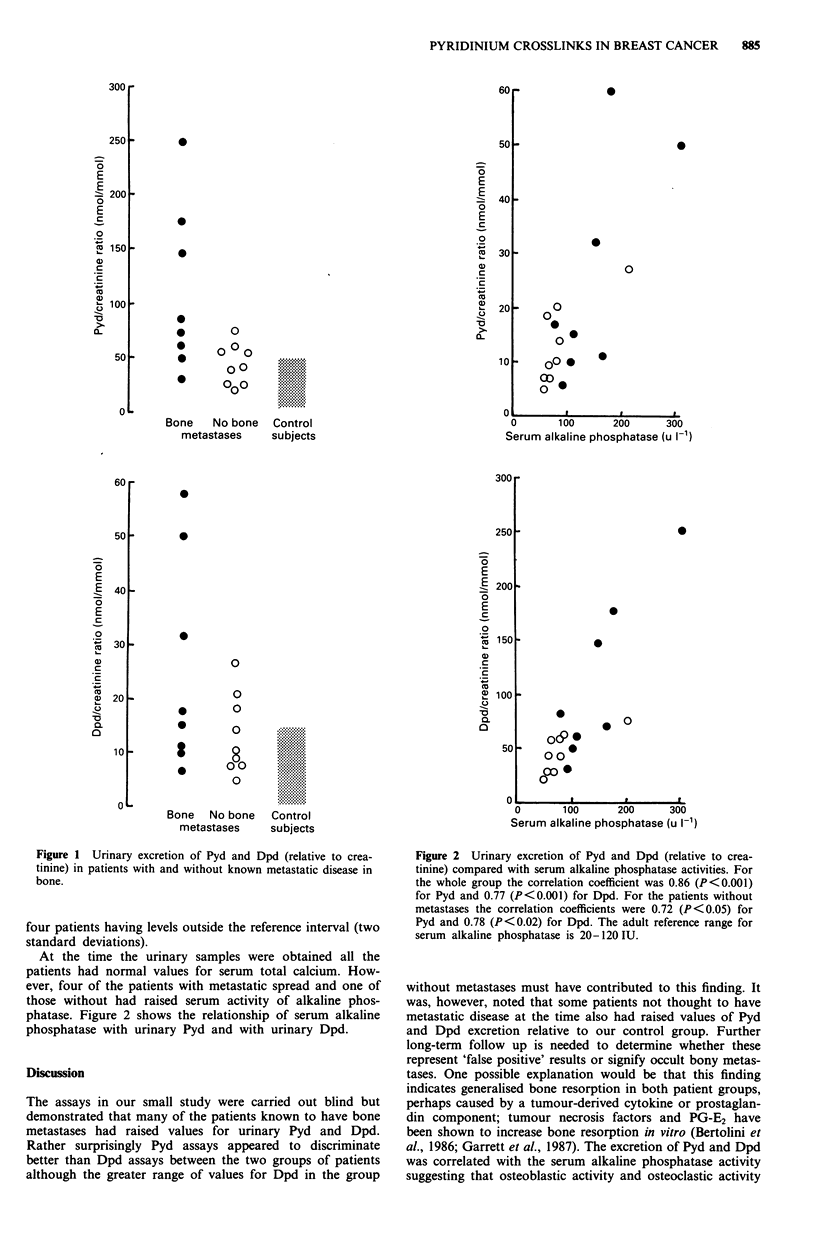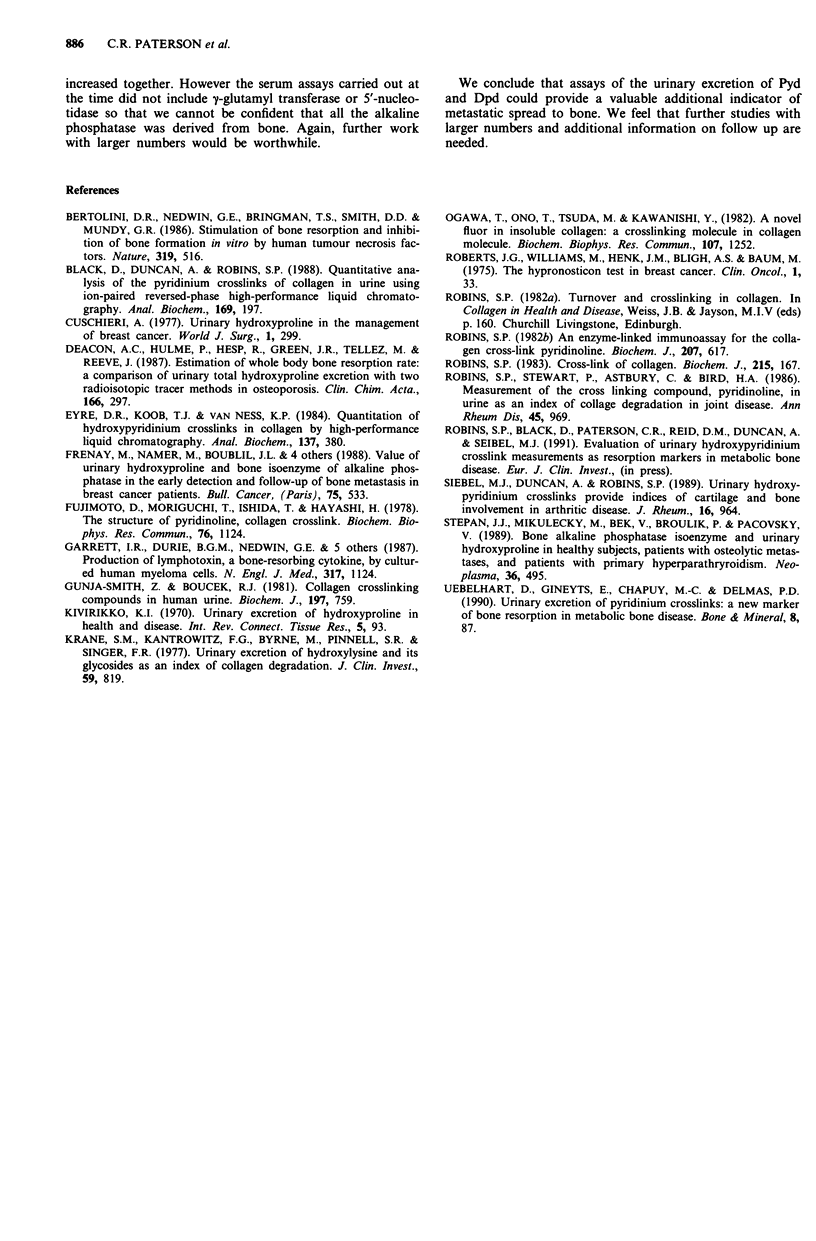# Pyridinium crosslinks as markers of bone resorption in patients with breast cancer.

**DOI:** 10.1038/bjc.1991.419

**Published:** 1991-11

**Authors:** C. R. Paterson, S. P. Robins, J. M. Horobin, P. E. Preece, A. Cuschieri

**Affiliations:** Department of Biochemical Medicine, University of Dundee, UK.

## Abstract

Collagen breakdown, and thus bone resorption, can now be assessed by measuring the urinary excretion of the collagen crosslinks, pyridinoline (Pyd) and deoxypyridinoline (Dpd). In a pilot study we measured Pyd and Dpd in 20 patients with breast cancer, ten with known bone metastases and ten with no recognised metastases in bone or elsewhere after 1 year's subsequent follow up. Eight out of the ten patients with metastases had crosslink excretion values higher than the reference interval, but so did some patients without known metastatic disease. For both crosslinks there was a clear correlation with serum alkaline phosphatase activity measured at about the same time. We consider that measurement of urinary collagen crosslink assays may have a place in the early detection of metastatic spread to bone.


*Br. J. Cancer* (1991), **64**, 884–886 © Macmillan Press Ltd., 1991

### Pyridinium crosslinks as markers of bone resorption in patients with breast cancer

C.R. Paterson[1], S.P. Robins[2], J.M. Horobin[3], P.E. Preece[3] & A. Cuschieri[3]

*Departments of [1]Biochemical Medicine and [3]Surgery, University of Dundee, Dundee DD1 4HN, and the [2]Rowett Research Institute, Aberdeen AB2 9SB, UK.*

**Summary** Collagen breakdown, and thus bone resorption, can now be assessed by measuring the urinary excretion of the collagen crosslinks, pyridinoline (Pyd) and deoxypyridinoline (Dpd). In a pilot study we measured Pyd and Dpd in 20 patients with breast cancer, ten with known bone metastases and ten with no recognised metastases in bone or elsewhere after 1 year's subsequent follow up. Eight out of the ten patients with metastases had crosslink excretion values higher than the reference interval, but so did some patients without known metastatic disease. For both crosslinks there was a clear correlation with serum alkaline phosphatase activity measured at about the same time. We consider that measurement of urinary collagen crosslink assays may have a place in the early detection of metastatic spread to bone.

Until recently the biochemical assessment of bone resorption has been limited by the lack of specificity of the methods available. Urinary hydroxyproline has been widely used. While greatly raised values are found in patients with a very high bone turnover such as Paget's disease, hydroxyproline assays have been less useful in demonstrating smaller changes in bone turnover, for example, in osteoporosis or primary hyperparathyroidism (Kivirikko, 1970; Stěpán *et al.*, 1989; Deacon *et al.*, 1987). The clinical usefulness of hydroxyproline is further limited by its derivation from tissues other than bone and from processes other than mature collagen turnover (Krane *et al.*, 1977; Robins, 1982*a*); it is also limited by the need for dietary restrictions to obtain the most reproducible results.

In the assessment of patients with breast malignancy, the place of hydroxyproline excretion has been explored; high values are seen in some patients with known bone metastases but many have values indistinguishable from normal (Roberts *et al.*, 1975; Cuschieri, 1977; Frenay *et al.*, 1988). The assay has proved to be of only limited value in the screening of patients in the follow up clinics.

Following the identification of collagen crosslink components in urine (Gunja-Smith & Boucek, 1981) an alternative method for assessing collagen breakdown was developed by using an immunoassay for pyridinoline (Robins, 1982*b*). Pyridinoline (Pyd) is a derivative of 3-hydroxyproline (Fujimoto *et al.*, 1978; Robins, 1983) and is present only in extracellular collagen fibrils. It is the principal crosslink of cartilage collagen and to a smaller extent of bone collagen; it is not found in skin collagen. High values of urinary Pyd have been demonstrated in patients with rheumatoid arthritis and osteoarthritis (Robins *et al.*, 1986; Seibel *et al.*, 1989).

An analogous crosslink, deoxypyridinoline (Dpd) has been identified in urine (Ogawa *et al.*, 1982); this component is derived almost exclusively from bone collagen (Eyre *et al.*, 1984). Recent work has shown that assays of Pyd and Dpd may be valuable markers of bone resorption in a wide range of metabolic bone diseases including Paget's disease, primary hyperparathyroidism, osteomalacia and even osteoporosis (Uebelhart *et al.*, 1990; Robins *et al.*, 1991).

We considered that the new assays might have a role in the assessment of patients with a history of breast carcinoma to provide early warning of bone metastases. We therefore carried out a pilot study of patients with previously recognised metastatic spread to bone and compared the results with those from a group of patients with no known metastases and who had been followed subsequently for not less than 1 year without evidence of metastatic disease in bone or elsewhere.

Correspondence: C.R. Paterson, Department of Biochemical Medicine, Ninewells Hospital and Medical School, Dundee DD1 9SY, UK.

Received 13 February 1991; and in revised form 13 June 1991.

### Patients and methods

All the patients were surveyed at the time the samples were obtained with a limited skeletal survey, including a chest X-ray, and with $^{99m}$Tc-polyphosphate scintigraphy. The ten patients with known metastatic disease in bone included two with metastatic disease also in the lungs and one who had a lesion thought to be metastatic in the contralateral breast. None of the patients had sclerotic metastases. At the end of the 1 year follow-up period, the patients without metastases were examined clinically and biochemically but the isotope scintigraphy was not repeated at this stage.

Urine samples were frozen within 6 h of collection and stored at −20°C. In an earlier study we had demonstrated that repeat assays on samples stored at −20°C showed no deterioration of the crosslinks (Robins *et al.*, 1991). The samples from the patients with and without metastatic spread were treated identically and the assays were carried out without knowledge of the diagnosis.

Analyses of the crosslinks were performed by HPLC (Black *et al.*, 1988; Seibel *et al.*, 1989). In all cases the urine samples (250 µl) were hydrolysed with an equal volume of concentrated HCl to release bound forms of the crosslinks. The results were expressed relative to the urinary creatinine concentration. Creatinine, calcium and alkaline phosphatase assays were done with standard automated procedures.

Statistical analyses were carried out using Student's *t*-test and linear regression with Pearson's correlation coefficients.

### Results

Figure 1 shows the urinary concentrations of Pyd and Dpd relative to creatinine in the two groups of patients. Values for a group of 118 healthy volunteers aged 21 to 74 (Seibel *et al.*, 1989) are shown for comparison. There were clear differences between the two groups of patients; for Pyd/Cr, the mean value (± s.d.) for the group with metastases (102.3 ± 67.1) was significantly higher ($P < 0.025$) than that for the group without metastases (44.5 ± 17.2). The corresponding values for Dpd (with metastases, 24.2 ± 17.4; without metastases, 12.3 ± 7.4) were statistically not significantly different. The values for the patients without known metastases were, however, significantly higher than those for the controls, with

**Figure 1** Urinary excretion of Pyd and Dpd (relative to creatinine) in patients with and without known metastatic disease in bone.

**Figure 2** Urinary excretion of Pyd and Dpd (relative to creatinine) compared with serum alkaline phosphatase activities. For the whole group the correlation coefficient was 0.86 ($P<0.001$) for Pyd and 0.77 ($P<0.001$) for Dpd. For the patients without metastases the correlation coefficients were 0.72 ($P<0.05$) for Pyd and 0.78 ($P<0.02$) for Dpd. The adult reference range for serum alkaline phosphatase is 20–120 IU.

four patients having levels outside the reference interval (two standard deviations).

At the time the urinary samples were obtained all the patients had normal values for serum total calcium. However, four of the patients with metastatic spread and one of those without had raised serum activity of alkaline phosphatase. Figure 2 shows the relationship of serum alkaline phosphatase with urinary Pyd and with urinary Dpd.

### Discussion

The assays in our small study were carried out blind but demonstrated that many of the patients known to have bone metastases had raised values for urinary Pyd and Dpd. Rather surprisingly Pyd assays appeared to discriminate better than Dpd assays between the two groups of patients although the greater range of values for Dpd in the group without metastases must have contributed to this finding. It was, however, noted that some patients not thought to have metastatic disease at the time also had raised values of Pyd and Dpd excretion relative to our control group. Further long-term follow up is needed to determine whether these represent 'false positive' results or signify occult bony metastases. One possible explanation would be that this finding indicates generalised bone resorption in both patient groups, perhaps caused by a tumour-derived cytokine or prostaglandin component; tumour necrosis factors and PG-$E_2$ have been shown to increase bone resorption *in vitro* (Bertolini *et al.*, 1986; Garrett *et al.*, 1987). The excretion of Pyd and Dpd was correlated with the serum alkaline phosphatase activity suggesting that osteoblastic activity and osteoclastic activity increased together. However the serum assays carried out at the time did not include γ-glutamyl transferase or 5′-nucleotidase so that we cannot be confident that all the alkaline phosphatase was derived from bone. Again, further work with larger numbers would be worthwhile.

We conclude that assays of the urinary excretion of Pyd and Dpd could provide a valuable additional indicator of metastatic spread to bone. We feel that further studies with larger numbers and additional information on follow up are needed.

### References

BERTOLINI, D.R., NEDWIN, G.E., BRINGMAN, T.S., SMITH, D.D. & MUNDY, G.R. (1986). Stimulation of bone resorption and inhibition of bone formation *in vitro* by human tumour necrosis factors. *Nature*, **319**, 516.

BLACK, D., DUNCAN, A. & ROBINS, S.P. (1988). Quantitative analysis of the pyridinium crosslinks of collagen in urine using ion-paired reversed-phase high-performance liquid chromatography. *Anal. Biochem.*, **169**, 197.

CUSCHIERI, A. (1977). Urinary hydroxyproline in the management of breast cancer. *World J. Surg.*, **1**, 299.

DEACON, A.C., HULME, P., HESP, R., GREEN, J.R., TELLEZ, M. & REEVE, J. (1987). Estimation of whole body bone resorption rate: a comparison of urinary total hydroxyproline excretion with two radioisotopic tracer methods in osteoporosis. *Clin. Chim. Acta.*, **166**, 297.

EYRE, D.R., KOOB, T.J. & VAN NESS, K.P. (1984). Quantitation of hydroxypyridinium crosslinks in collagen by high-performance liquid chromatography. *Anal. Biochem.*, **137**, 380.

FRENAY, M., NAMER, M., BOUBLIL, J.L. & 4 others (1988). Value of urinary hydroxyproline and bone isoenzyme of alkaline phosphatase in the early detection and follow-up of bone metastasis in breast cancer patients. *Bull. Cancer, (Paris)*, **75**, 533.

FUJIMOTO, D., MORIGUCHI, T., ISHIDA, T. & HAYASHI, H. (1978). The structure of pyridinoline, collagen crosslink. *Biochem. Biophys. Res. Commun.*, **76**, 1124.

GARRETT, I.R., DURIE, B.G.M., NEDWIN, G.E. & 5 others (1987). Production of lymphotoxin, a bone-resorbing cytokine, by cultured human myeloma cells. *N. Engl. J. Med.*, **317**, 1124.

GUNJA-SMITH, Z. & BOUCEK, R.J. (1981). Collagen crosslinking compounds in human urine. *Biochem. J.*, **197**, 759.

KIVIRIKKO, K.I. (1970). Urinary excretion of hydroxyproline in health and disease. *Int. Rev. Connect. Tissue Res.*, **5**, 93.

KRANE, S.M., KANTROWITZ, F.G., BYRNE, M., PINNELL, S.R. & SINGER, F.R. (1977). Urinary excretion of hydroxylysine and its glycosides as an index of collagen degradation. *J. Clin. Invest.*, **59**, 819.

OGAWA, T., ONO, T., TSUDA, M. & KAWANISHI, Y., (1982). A novel fluor in insoluble collagen: a crosslinking molecule in collagen molecule. *Biochem. Biophys. Res. Commun.*, **107**, 1252.

ROBERTS, J.G., WILLIAMS, M., HENK, J.M., BLIGH, A.S. & BAUM, M. (1975). The hypronosticon test in breast cancer. *Clin. Oncol.*, **1**, 33.

ROBINS, S.P. (1982*a*). Turnover and crosslinking in collagen. In *Collagen in Health and Disease*, Weiss, J.B. & Jayson, M.I.V (eds) p. 160. Churchill Livingstone, Edinburgh.

ROBINS, S.P. (1982*b*) An enzyme-linked immunoassay for the collagen cross-link pyridinoline. *Biochem. J.*, **207**, 617.

ROBINS, S.P. (1983). Cross-link of collagen. *Biochem. J.*, **215**, 167.

ROBINS, S.P., STEWART, P., ASTBURY, C. & BIRD, H.A. (1986). Measurement of the cross linking compound, pyridinoline, in urine as an index of collage degradation in joint disease. *Ann Rheum Dis*, **45**, 969.

ROBINS, S.P., BLACK, D., PATERSON, C.R., REID, D.M., DUNCAN, A. & SEIBEL, M.J. (1991). Evaluation of urinary hydroxypyridinium crosslink measurements as resorption markers in metabolic bone disease. *Eur. J. Clin. Invest.*, (in press).

SIEBEL, M.J., DUNCAN, A. & ROBINS, S.P. (1989). Urinary hydroxypyridinium crosslinks provide indices of cartilage and bone involvement in arthritic disease. *J. Rheum.*, **16**, 964.

STEPAN, J.J., MIKULECKY, M., BEK, V., BROULIK, P. & PACOVSKY, V. (1989). Bone alkaline phosphatase isoenzyme and urinary hydroxyproline in healthy subjects, patients with osteolytic metastases, and patients with primary hyperparathryroidism. *Neoplasma*, **36**, 495.

UEBELHART, D., GINEYTS, E., CHAPUY, M.-C. & DELMAS, P.D. (1990). Urinary excretion of pyridinium crosslinks: a new marker of bone resorption in metabolic bone disease. *Bone & Mineral*, **8**, 87.